# In Vitro Biocompatibility of Calcium Silicate-Based Materials for Retrograde Endodontic Treatment Under Different Setting Conditions

**DOI:** 10.3390/jfb17030124

**Published:** 2026-03-04

**Authors:** Kremena Markova, Neshka Manchorova-Veleva, Veselina Todorova, Lyubomir Vangelov, Desislava Petkova

**Affiliations:** Department of Operative Dentistry and Endodontics, Faculty of Dental Medicine, Medical University of Plovdiv, 4002 Plovdiv, Bulgaria; neshka.manchorova@mu-plovdiv.bg (N.M.-V.); lyubomir.vangelov@mu-polvdiv.bg (L.V.); desislava.petkova@mu-plovdiv.bg (D.P.)

**Keywords:** calcium silicate materials, endodontic biomaterials, eluate-based cytotoxicity, cell viability, fibroblasts, blood contamination, retrograde endodontic treatment, setting conditions

## Abstract

Background: Calcium silicate-based materials are widely used in retrograde endodontic treatment due to their bioactivity and favorable biological properties. The environmental conditions during setting and the time-dependent release of soluble components may influence cellular responses; however, these factors remain insufficiently investigated. Aim: This in vitro study evaluated the cellular response to three calcium silicate-based materials—MTA+, Biodentine, and NeoPUTTY—after setting under different environmental conditions. Materials and Methods: Cylindrical specimens were allowed to set under three conditions: dry environment, phosphate-buffered saline (PBS), and human blood. Eluates obtained after 1, 3, and 5 days were applied to human BJ fibroblasts. Cell viability, based on metabolic activity measured using the AlamarBlue assay, was evaluated at 48 and 96 h. Biocompatibility was inferred from cell viability, reflecting eluate-mediated effects rather than direct material–cell contact. Results: Cell viability was influenced by both the setting environment and eluate maturation time. PBS-set materials showed variable cellular responses, with high viability at early time points but marked decreases at 96 h for some MTA+ and NeoPUTTY groups. Biodentine demonstrated the most stable cellular response across all conditions. Materials set in blood produced cellular responses comparable to those observed for PBS and dry conditions, with no statistically significant overall reduction in cell viability. Conclusions: Within the limitations of this in vitro eluate-based model, blood exposure during setting had a minimal influence on the cell viability to the tested materials. Among the evaluated materials, Biodentine exhibited the most stable biological profile. These findings reflect time-dependent, eluate-mediated cellular effects and should be interpreted with caution when extrapolating to clinical conditions.

## 1. Introduction

Calcium silicate-based biomaterials play a central role in modern endodontics due to their favorable physicochemical properties, sealing ability, and biological performance. Since the introduction of mineral trioxide aggregate (MTA), these materials have been widely used for retrograde root-end fillings, perforation repair, pulp capping, apexification, and regenerative procedures [1]. Their clinical success is largely attributed to their ability to interact positively with surrounding tissues, promote mineralized tissue formation, and maintain dimensional stability in moist environments [2].

Biocompatibility is a key requirement for endodontic biomaterials and is defined as the ability of a material to perform its intended function without causing adverse local or systemic effects while supporting appropriate biological responses [3,4]. During surgical endodontic procedures, these materials come into direct contact with periapical connective tissues, where cellular responses such as adhesion, proliferation, migration, and cell viability are critical for healing and tissue regeneration [5].

Fibroblasts represent one of the main cell populations involved in periapical wound healing and extracellular matrix formation. They play an essential role in tissue repair, inflammatory modulation, and structural reorganization following surgical intervention [6]. For this reason, fibroblast-based in vitro models are widely used and recommended for cytocompatibility testing of dental biomaterials according to international standards [7,8].

The biological behavior of calcium silicate-based materials is closely related to their hydration reactions. Upon contact with moisture, these materials form calcium silicate hydrate and calcium hydroxide, leading to the release of calcium ions and hydroxyl groups and an increase in local pH [9]. These physicochemical changes are associated with antibacterial effects and bioactivity, including the formation of apatite-like deposits in phosphate-containing environments [10,11]. At the same time, elevated ionic concentrations and alkaline conditions may influence cellular metabolism and proliferation under in vitro conditions.

In clinical practice, retrograde filling materials rarely set under ideal laboratory conditions. Instead, they are frequently exposed to physiological fluids or blood during placement, particularly in apical surgery and perforation repair [12]. Blood contamination has been shown to affect setting reactions, surface characteristics, sealing ability, and bioactivity of calcium silicate materials [13,14,15]. However, its influence on the biological effects of material eluates remains insufficiently investigated.

Most in vitro studies evaluate cytocompatibility using eluates obtained from materials set under standardized laboratory conditions and often subjected to dilution protocols [16,17]. Although this approach allows assessment of dose–response relationships, it may not adequately reflect clinical situations where materials set in the presence of tissue fluids or blood. In addition, the composition of material eluates changes over time as hydration reactions progress and ion release continues [18,19,20]. Early eluates may contain higher concentrations of reactive components, whereas later eluates reflect a more stabilized release profile, which may influence cellular responses differently [21,22,23].

Newer calcium silicate-based materials have been developed to improve handling characteristics and clinical performance. Biodentine contains calcium chloride as a setting accelerator and has demonstrated favorable biological properties, while premixed putty formulations such as NeoPUTTY aim to provide consistent composition and easier clinical application [24]. However, comparative data regarding their biological behavior after setting under clinically relevant environmental conditions remain limited.

Therefore, the aim of the present study was to evaluate the biocompatibility of three calcium silicate-based endodontic materials—MTA+, Biodentine, and NeoPUTTY—using eluates obtained after setting under different environmental conditions (dry, phosphate-buffered saline, and human blood).

It was hypothesized that both the setting environment and the duration of eluate extraction would influence fibroblast viability.

By assessing time-dependent eluates from materials set in the presence of blood, this study seeks to provide additional information on material–cell interactions under conditions that more closely simulate the clinical environment.

## 2. Materials and Methods

The study was approved by the Institutional Ethics Committee of Medical University of Plovdiv, Bulgaria (Protocol number 2/date of approval 9 March 2023 and final decision number P-663/21 March 2023).

Three commercially available calcium silicate-based endodontic biomaterials were evaluated in the present study: MTA+ (Cerkamed, Stalowa Wola, Poland), Biodentine (Septodont, Saint-Maur-des-Fossés, France), and NeoPUTTY (NuSmile, Houston, TX, USA). A zinc oxide–eugenol cement (ZnOE) was included as a cytotoxic reference material due to its well-documented in vitro cytotoxicity. All materials were handled strictly according to the manufacturers’ instructions to ensure clinical relevance and reproducibility.

To obtain comprehensive information on the influence of setting conditions on the biocompatibility of the tested materials, a cytotoxicity and cell viability assay was employed. The biological model used was the human fibroblast cell line BJ (CRL-2522™), an established cell line originally isolated from the foreskin tissue of a neonatal male donor and obtained from the American Type Culture Collection (ATCC, Manassas, VA, USA), passages 47 and 52.

### 2.1. Preparation of Test Specimens and Eluate Extraction

Cylindrical specimens were prepared from each calcium silicate-based material (MTA+, Biodentine, and NeoPUTTY), with a total of 33 specimens per material (5 mm in diameter and 2 mm in height), in accordance with the manufacturers’ instructions. ZnOE specimens (n = 11) were also prepared. The specimen dimensions were selected to provide a standardized and clinically relevant material volume while ensuring reproducible surface characteristics. The extraction procedure followed the recommendations of ISO 10993-12 [25], maintaining an appropriate material surface area-to-extraction medium ratio (approximately 3 cm^2^/mL). This standardized ratio ensured comparable ion release conditions and allowed meaningful interpretation of eluate-mediated biological effects.

The materials were placed into standardized cylindrical moulds made of inert non-reactive plastic to ensure uniform specimen geometry and to prevent interaction between the mould material and the setting biomaterials. The specimens of the investigated calcium-silicate materials were divided into three subgroups (n = 11 per condition) based on the setting environment:

• Group I: Set in a dry environment (n = 11 for each material), Condition 1 (C1).
• Group II: Set in phosphate-buffered saline (PBS) (n = 11 for each material), Condition 2 (C2).
• Group III: Set in human blood (n = 11 for each material), Condition 3 (C3).

The blood was obtained from a healthy human volunteer (one of the authors, K.M.) after informed consent, in accordance with the approval of the institutional ethics committee. The blood was collected in sterile Vacutainer tubes containing heparin as an anticoagulant to prevent coagulation.

The ZnOE specimens were allowed to set only under dry conditions according to the manufacturer’s instructions, as this material is conventionally mixed and used in a moisture-free environment. It was included as a cytotoxic reference rather than for comparison of environmental setting effects.

After placement in the respective environments, the specimens were stored in an incubator at 37 °C and 95% humidity for 48 h. The prepared specimens were sterilized with UV light for 30 min, after which three samples of each material, set under each of the three conditions, were placed in a sterile Eppendorf microtube to which 1 mL of cell culture medium DMEM (Dulbecco’s Modified Eagle’s Medium—high glucose, 4.5 g/L) was added, following ISO 10993-5 [26]. The ratio of material surface area to the volume of the extraction carrier was maintained. These samples were then incubated for 1, 3, and 5 days at 37 °C, 5% CO_2_ saturation, and 85% humidity. The selected time points were intended to reflect the time-dependent changes in material hydration and ion release, representing early (1 day), intermediate (3 days), and later (5 days) stages of eluate maturation.

**Figure 1:**
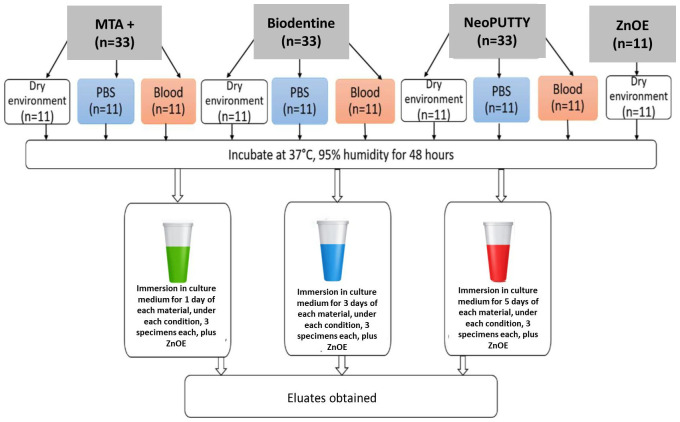
Eluate preparation protocol. Reprinted with permission from Ref. [27].

After the respective periods, the obtained eluates (modified media) were withdrawn from the Eppendorf test tubes and stored at 4 °C. Eluates were obtained from three materials cured under three different conditions (dry environment, phosphate-buffered saline (PBS) and blood) and incubated in cell culture medium for 1, 3, and 5 days, resulting in a total of 27 different eluates. The ZnOE samples were also incubated for 1, 3, and 5 days, and the obtained eluates were used for cytotoxic control reference (Figure 1). The total number of eluates was 30.

To facilitate the experimental procedure as well as the recording and presentation of the results, the following coding system was applied:

• M1—MTA+.
• M2—Biodentine.
• M3—NeoPUTTY.
• C1—Material set in a dry environment.
• C2—Material set in phosphate-buffered saline (PBS).
• C3—Material set in blood.
• D1—Eluate obtained after 1 day of incubation in cell culture medium.
• D3—Eluate obtained after 3 days of incubation in cell culture medium.
• D5—Eluate obtained after 5 days of incubation in cell culture medium.

### 2.2. Cell Culture Preparation

The experiments were conducted using cultured human fibroblast cells. Cells from the BJ (CRL-2522™; ATCC, Manassas, VA, USA), a stable fibroblast cell line isolated from human foreskin, were used, passages 47 and 52 (passage number), which were selected to ensure stable growth characteristics and reproducible metabolic activity. Cells were stored in 1.8 mL cryovials at −150 °C.

The BJ fibroblast cell line was selected because human dermal fibroblasts exhibit biological behavior comparable to fibroblasts present in periapical connective tissues, including sensitivity to changes in extracellular pH, ionic composition, and released material components. Compared with primary periodontal ligament or apical papilla cells, the use of a well-characterized continuous cell line provides greater experimental reproducibility, phenotypic stability, and reduced donor-to-donor variability, which is particularly important for standardized cytotoxicity testing.

A sterile cell culture medium, DMEM (Dulbecco’s Modified Eagle’s Medium—high glucose, 4.5 g/L), supplemented with 10% fetal bovine serum (FBS), 1% antibiotic-antimycotic solution, was prepared and stored at 4 °C. Before each use, the cell culture medium was warmed in a water bath to 37 °C.

After thawing, the cells were transferred to two T-25 flasks and cultured at 37 °C, 5% CO_2_, and 95% humidity.

After subsequent passaging and expansion, the fibroblasts were transferred into a 96-well plate for the cell viability assay.

### 2.3. Cell Viability Assay

Twenty-four hours after seeding the cells into a 96-well plate at a density of 5 × 10^4^ cells per well, the culture medium was removed and 150 µL of each obtained eluate (n = 30) was added. Each eluate was applied to three parallel wells (technical replicates), and the mean value was used for further analysis.

Cells cultured in standard culture medium without eluate exposure served as the control group (non-toxic; untreated cells).

Considering the hydrophilic nature of the investigated materials, which can release ionic components and influence intracellular enzymatic activity, a test measuring mitochondrial dehydrogenase activity was performed using the redox indicator dye AlamarBlue Cell Viability Reagent (Thermo Fisher Scientific, Waltham, MA, USA).

After 48 h, the eluates were removed and the cells were treated with AlamarBlue for 4 h. This reagent contains the permeable, non-toxic, water-soluble, and fluorescent blue indicator dye resazurin. AlamarBlue serves as a direct indicator of cell health by detecting the level of oxidation during cellular respiration, thereby quantitatively measuring cell viability and cytotoxicity. As a redox indicator dye, AlamarBlue allows indirect quantitative determination of cell number based on metabolic reactions intrinsic to living cells and has the advantage of detecting only viable cells.

Cell viability was determined by calculating the difference in resazurin reduction in treated cells compared with control wells. Cytotoxic responses were classified as severe (<30%), moderate (30–60%), mild (60–90%), or non-cytotoxic (>90%) based on activity relative to values obtained from control wells.

Measurements were performed using a FLUOstar OPTIMA microplate reader (BMG LABTECH, Ortenberg, Germany), which records fluorescence intensity and light absorbance at wavelengths between 570 and 600 nm.

Cell viability was calculated as a percentage using the formula:

100 (a − b)/c,

where a and b represent the fluorescence intensity values of the tested and background wells, respectively, and c is the mean fluorescence intensity value of the control wells.

Measurements were conducted for each material, setting condition, and incubation period. After the initial evaluation, the cells were treated again with the corresponding eluates for an additional 48 h, and the measurements were repeated following the same protocol.

Cell viability was evaluated after 48 h and following an additional 48 h exposure (total 96 h) in order to assess both early and delayed cellular responses to material eluates. The initial 48 h time point reflects acute cytotoxic or metabolic effects associated with higher concentrations of released components, whereas the 96 h evaluation allows detection of delayed effects, cellular recovery, or cumulative responses resulting from prolonged exposure. This approach provides a more comprehensive assessment of time-dependent cytocompatibility of calcium silicate-based materials.

### 2.4. Statistical Analysis

Statistical analysis was performed using IBM SPSS Statistics (v.25) at a significance level of *p* < 0.05. Descriptive statistics were calculated as mean values, which are presented in the figures and text.

Normality of data distribution within groups was assessed using the Kolmogorov–Smirnov test with Lilliefors significance correction and the Shapiro–Wilk test. To evaluate the effects of material type, setting conditions, incubation day, and measurement time on the assessed parameters, a mixed repeated-measures analysis of variance (Mixed Repeated ANOVA) was applied, with time as a within-subject factor and material, setting condition, and incubation day as between-subject factors.

When normal distribution was confirmed for all compared groups, the effect of time was analyzed using repeated-measures ANOVA or the Greenhouse–Geisser correction. If at least one group deviated from normality, the Friedman test was applied. For the evaluation of material and setting condition effects, one-way ANOVA or the Brown–Forsythe test (robust tests of equality of means) was used when normality was present; otherwise, the Kruskal–Wallis test was applied.

In cases of statistically significant differences identified by parametric methods, pairwise comparisons were performed using the Bonferroni test for time-related comparisons and either the Bonferroni or Games–Howell test for material and setting condition comparisons, depending on variance homogeneity. When non-parametric tests indicated statistically significant differences, pairwise comparisons were conducted using the Wilcoxon signed-rank test for time-related comparisons and the Mann–Whitney U test for material and setting condition comparisons.

The results obtained from non-parametric tests consistently corresponded with those from their parametric counterparts, despite deviations from normal distribution.

## 3. Results

Comparison of the results obtained after 48 and 96 h of treatment revealed statistically significant differences for all eluates derived from NeoPUTTY, regardless of the setting environment and incubation period. Similar findings were observed for MTA+, with the exception of specimens set in PBS and incubated for 1 day. In contrast, Biodentine showed minimal differences between the two evaluation time points. A statistically significant difference was detected only for the 3-day eluates obtained from blood-set specimens and for Biodentine specimens set in PBS and incubated for 5 days (*p* < 0.05). Overall, cell viability values at 96 h were generally higher for Biodentine than for MTA+ and NeoPUTTY.

**Figure 2:**
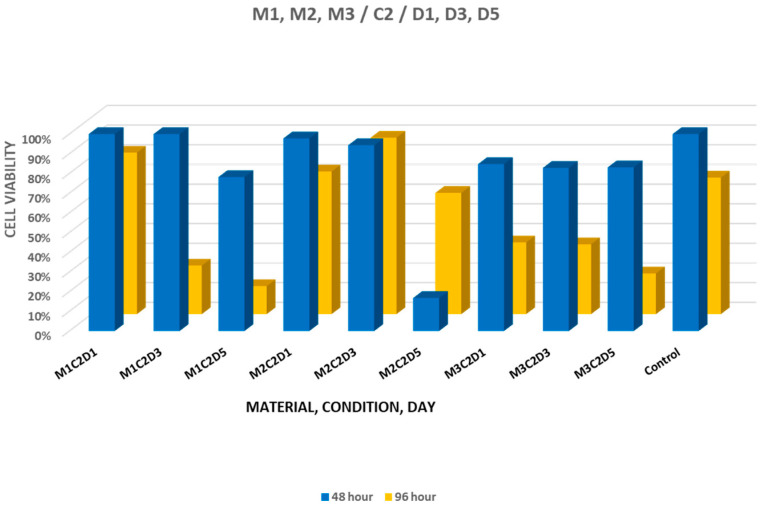
Cell viability after treatment with 1-, 3-, and 5-day eluates of MTA+ (M1), Biodentine (M2), and NeoPUTTY (M3) set in PBS (C2).

When comparing cell viability at 48 and 96 h under the influence of eluates derived from materials set in PBS, cell viability values observed at 48 h ranged between 78.20% and 100.04% for MTA+, 16.76% and 97.80% for Biodentine, and 82.81% and 84.73% for NeoPUTTY. A reduced value was observed for the 5-day Biodentine eluate at 48 h, indicating a transient decrease in cell viability. At 96 h, viability ranged between 14.18% and 81.96% for MTA+, 61.54% and 89.54% for Biodentine, and 20.66% and 36.41% for NeoPUTTY (Figure 2). Notably, Biodentine showed recovery to moderate-to-high viability levels at 96 h. A statistically significant difference between the two evaluation time points was detected for selected eluates (*p* < 0.05), including the 5-day Biodentine eluate. For NeoPUTTY set in PBS, cell viability at 48 h did not differ significantly among incubation days (*p* = 0.976). At 96 h, overall viability values were lower compared with 48 h (Figure 2).

**Figure 3:**
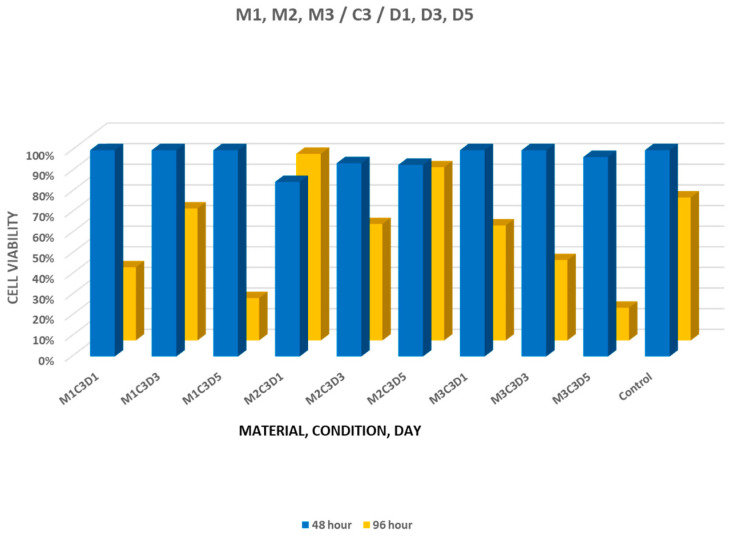
Cell viability after treatment with 1-, 3-, and 5-day eluates of MTA+ (M1), Biodentine (M2), and NeoPUTTY (M3) set in blood (C3).

At 48 h, materials set in blood demonstrated generally high cell viability, with values comparable to those of the untreated control group. When comparing materials set in blood, statistically significant differences at 96 h were observed for the 3-day eluates of MTA+ and Biodentine and for the 1-day eluates of NeoPUTTY (*p* < 0.05). For blood-set specimens, the highest viability values were 63.98% for MTA+ and 55.73% for NeoPUTTY, while Biodentine showed values between 56.43% and 90.46% (Figure 3).

Analysis of the effect of incubation time showed a statistically significant difference at the first evaluation time point only for NeoPUTTY (*p* = 0.025), whereas at 96 h, a statistically significant difference was also detected for MTA+. No statistically significant influence of incubation time was observed for Biodentine.

**Figure 4:**
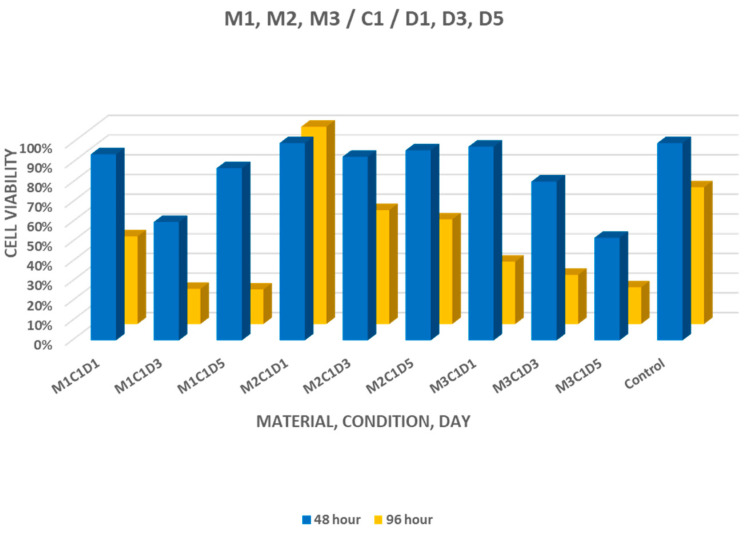
Cell viability after treatment with 1-, 3-, and 5-day eluates of MTA+ (M1), Biodentine (M2), and NeoPUTTY (M3) set in a dry environment (C1).

For materials set under dry conditions, cell viability at 48 h ranged between 59.98% and 94.30% for MTA+, 93.06% and 99.98% for Biodentine, and 51.97% and 97.16% for NeoPUTTY. At 96 h, the values ranged between 17.49% and 44.43% for MTA+, 53.03% and 99.67% for Biodentine, and 18.58% and 31.60% for NeoPUTTY (Figure 4). Statistically significant differences between setting environments were detected for selected eluates of MTA+ and Biodentine (*p* < 0.05).

A statistically significant difference was observed only between the viability of cells treated with eluates from MTA+ set in blood and incubated for 3 days and those treated with eluates from the same material set in a dry environment. A similar statistically significant difference was detected for Biodentine eluates set in a dry environment and in PBS and incubated for 3 days (*p* < 0.05).

**Figure 5:**
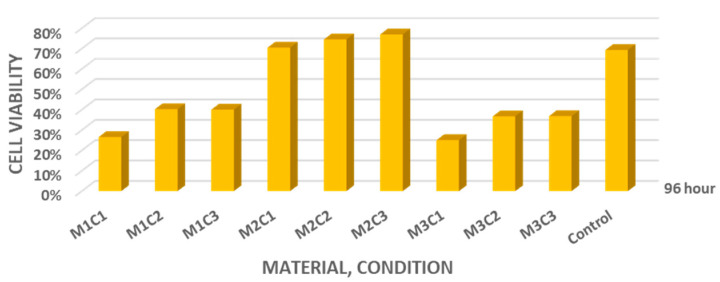
Average cell viability values after treatment with 1-, 3-, and 5-day eluates of MTA+ (M1), Biodentine (M2), and NeoPUTTY (M3) set under dry conditions (C1), in PBS (C2), and in blood (C3).

Overall, all investigated materials—MTA+, Biodentine, and NeoPUTTY—demonstrated variable cell viability compared with the control group at 96 h. The average cell viability values at 96 h were 26.56% (dry), 40.26% (PBS), and 40.00% (blood) for MTA+; 70.47%, 74.48%, and 76.95% for Biodentine; and 25.02%, 36.74%, and 36.85% for NeoPUTTY, respectively (Figure 5). Among the tested materials, Biodentine showed the highest overall cell viability.

The cytotoxic reference material (ZnOE), set under dry conditions, showed markedly reduced cell viability, with mean values of approximately 16.78% at 48 h and 3.08% at 96 h, indicating substantially lower viability compared with untreated control cells and the tested materials.

## 4. Discussion

Biocompatibility is a critical requirement for retrograde filling materials, as these materials come into direct or indirect contact with periapical tissues and influence cellular responses essential for healing. Cytotoxicity assays based on cell viability are widely used to assess the biological safety of calcium silicate-based materials and to estimate their potential impact on reparative processes [28,29].

In the present study, Biodentine demonstrated the most stable cellular response across different setting conditions and eluate maturation periods. These findings are consistent with previous reports describing favorable cytocompatibility and biological behavior of Biodentine in fibroblast and stem cell models [17,30,31,32,33]. Several studies have shown that initial reductions in cell viability after exposure to calcium silicate materials may be followed by recovery over time, reflecting cellular adaptation to the physicochemical environment rather than persistent cytotoxicity [8,34].

A reduction in cell viability does not necessarily indicate toxic effects alone. Exposure to calcium silicate eluates may alter cellular proliferation due to increased extracellular calcium concentration and alkaline pH. Elevated Ca^2+^ levels can modulate intracellular signaling pathways and mitochondrial activity, leading either to transient metabolic suppression or to an induction of differentiation, which is associated with reduced proliferative activity [35,36,37,38,39,40,41]. Therefore, decreased cell viability observed in vitro should be interpreted cautiously, as it may reflect bioactivity-related cellular modulation rather than irreversible cytotoxic damage.

The influence of pH represents another important factor. Calcium silicate materials release hydroxyl ions during hydration, resulting in an alkaline environment that may temporarily inhibit fibroblast proliferation, as optimal growth occurs within a narrow physiological pH range [42,43,44]. In some groups, particularly for Biodentine, recovery or stabilization of cell viability at later time points suggests transient rather than sustained biological stress.

For NeoPUTTY and MTA+, moderate reductions in cell viability were observed at specific time points, particularly after exposure to more mature eluates. Similar findings have been reported in previous studies [35,36,37,38,39,40,41] and have been attributed to differences in material composition, including radiopacifying agents and trace metallic impurities [45,46,47,48,49,50,51,52,53,54]. The cytotoxic potential of bismuth oxide as a radiopacifier has been evaluated in various cell lines, with results indicating that cells are able to recover over time without persistent differences in viability [49,50]. In contrast, studies assessing the toxicity of zirconium oxide, used as the radiopacifying agent in Biodentine, have reported more favorable outcomes and an absence of cytotoxic effects [51,55,56,57]. Another explanation for Biodentine’s low cytotoxicity may be the use of active biosilicate technology, which eliminates metallic impurities typically found in MTA cements [57]. Biodentine has been shown to release significantly higher levels of calcium and silicon ions compared with MTA [58,59], suggesting that increased calcium and silicon release may contribute to its improved biocompatibility.

Another proposed explanation for reduced cell viability is the presence of unpurified mixtures containing low concentrations of metallic impurities. Researchers have detected toxic heavy metals, including arsenic, chromium, and lead, in MTA eluates [52,53,54]. The liquid component of Biodentine contains calcium chloride, added as a setting accelerator, which appears to contribute to its biocompatibility [60].

Conflicting results have been reported regarding the comparative biocompatibility of Biodentine and MTA. Some studies describe superior performance of Biodentine [60,61,62], whereas others report similar biological responses between the two materials [8,17]. These discrepancies may be explained by differences in experimental design, including cell type, assay method [63,64,65], eluate preparation, dilution protocols [45,48,64,66], and observation period [63,64,65,66,67]. Most studies conclude within 48 or 72 h [67]; in the present study, cell viability was assessed at 96 h, which may account for some of the differences observed relative to other reports. The variability inherent in in vitro models highlights the importance of standardized testing conditions when comparing materials.

One of the main objectives of this study was to evaluate the effect of the setting environment, particularly blood contamination, on the biological response to material eluates. The results demonstrated that specimens set in blood produced cell viability values comparable to those obtained for materials set in PBS and dry conditions. These findings indicate that exposure to blood during the setting phase did not significantly impair eluate-mediated cytocompatibility under the conditions of the present study.

From a clinical perspective, this observation is relevant because retrograde filling materials frequently come into contact with blood during apical surgery, perforation repair, and other endodontic procedures, where complete isolation is difficult to achieve. Blood contamination has been reported to influence the hydration reaction, microstructure, surface morphology, mechanical properties, and sealing ability of calcium silicate-based materials [68,69,70,71,72,73,74]. Interaction with blood components may interfere with crystal formation and alter the material surface, potentially affecting the release profile of calcium ions and other soluble products.

Despite these reported physicochemical effects, the present results suggest that such changes did not translate into a marked alteration of the biological response to material eluates. Several factors may contribute to this finding. Blood proteins and organic components may form a surface layer that modifies, but does not necessarily increase, the release of cytotoxic substances. In addition, the buffering capacity of blood and its complex composition may reduce extreme pH changes or dilute reactive components released during the early stages of material hydration. As a result, the overall cellular response to the eluates remained comparable to that observed for specimens set in physiological solution.

The available literature addressing the biological effects of blood-contaminated calcium silicate materials is limited and inconsistent, with most studies focusing primarily on physical or sealing properties rather than cellular responses [61,74]. Therefore, the present findings contribute additional evidence suggesting that, although blood contamination may influence material characteristics, its impact on eluate-mediated cytocompatibility appears to be limited.

In addition, eluate-based models evaluate the effects of diffusible components rather than direct surface–cell interactions, which may explain differences compared with studies using direct-contact approaches.

It should be emphasized that in vitro conditions do not replicate the complex biological environment present in vivo. Cell culture systems lack vascularization, immune responses, and physiological mechanisms responsible for dilution, clearance, and tissue adaptation [75,76]. Therefore, the results of eluate-based assays should be interpreted as preliminary indicators of cytocompatibility rather than direct predictors of clinical performance. These limitations of in vitro cytotoxicity testing, particularly for eluate-based cell viability assays, have been highlighted in recent systematic reviews, which emphasize that such models provide preliminary biological screening but cannot fully predict clinical performance [77].

Overall, the present findings suggest that the tested calcium silicate-based materials exhibit favourable cytocompatibility under the conditions of this in vitro eluate-based model, with Biodentine demonstrating the most stable biological profile and blood contamination showing minimal influence on cellular responses.

## 5. Limitations and Future Research

The present study has several limitations that should be considered when interpreting the findings. First, the investigation was conducted under in vitro conditions using a single fibroblast cell line. Although fibroblasts are biologically relevant for evaluating material–cell interactions in periapical tissues, in vitro models cannot fully replicate the complex physiological environment, including immune responses, vascularization, and dynamic fluid exchange present in vivo. Consequently, extrapolation of these results to clinical conditions should be made with caution.

Second, only cell viability was assessed as an indicator of biocompatibility. While this parameter provides valuable information regarding cytotoxic effects, it does not capture other important biological processes, such as cell differentiation, inflammatory signaling, gene expression, or mineralization potential. Future studies incorporating additional assays, including osteogenic markers, inflammatory cytokine profiling, and gene expression analysis, would provide a more comprehensive evaluation of material bioactivity.

Third, the study evaluated a limited number of setting environments and incubation periods. Although dry conditions, PBS, and blood were selected to reflect clinically relevant scenarios, other variables, such as prolonged aging, repeated fluid exchange, or interaction with saliva and inflammatory exudates, may further influence material behavior and cellular responses. Long-term studies examining eluate composition and biological effects over extended time periods are therefore warranted.

Finally, the present investigation focused on eluate-mediated effects rather than direct cell–material contact. While eluate-based testing is recommended by international standards and allows standardized comparison, complementary direct-contact models and three-dimensional culture systems may better simulate clinical conditions. Future research should also include in vivo studies to validate the in vitro findings and to assess tissue-level responses under clinically relevant conditions.

The present study represents one component of a broader research project investigating the influence of setting conditions on the physicochemical properties and biological performance of calcium silicate-based materials. The current work focused on eluate-mediated cytocompatibility, while complementary physicochemical analyses and additional experimental models are planned for future investigations.

## 6. Conclusions

Within the limitations of this in vitro eluate-based study, blood contamination during setting did not result in a significant overall reduction in the cell viability of the tested calcium silicate-based materials. Materials set in blood demonstrated cellular responses comparable to those observed under phosphate-buffered saline and dry conditions.

All three investigated materials—MTA+, Biodentine, and NeoPUTTY—showed variable cell viability depending on the setting condition and eluate maturation time. MTA+ and NeoPUTTY demonstrated moderate to marked reductions in cell viability under certain conditions, whereas Biodentine exhibited the most stable cellular response and the lowest sensitivity to the setting environment.

These findings reflect indirect, eluate-mediated effects and should be interpreted with caution. Further studies using direct-contact experimental models and in vivo investigations are necessary to confirm the clinical relevance of the observed biological responses.

## Data Availability

The authors confirm that the data supporting the findings of this study are available within the article. Further inquiries can be directed to the corresponding author.

## Abbreviations

The following abbreviations are used in this manuscript:

ANOVA	Analysis of variance
ATCC	American Type Culture Collection
BJ	Human fibroblast cell line (CRL-2522™)
Ca^2+^	Calcium ions
CO_2_	Carbon dioxide
DMEM	Dulbecco’s Modified Eagle’s Medium (high glucose, 4.5 g/L)
FBS	Fetal bovine serum
ISO	International Organization for Standardization
MTA	Mineral trioxide aggregate
PBS	Phosphate-buffered saline
UV	Ultraviolet
ZnOE	Zinc oxide eugenol cement
